# Long-term ecological and evolutionary dynamics in the gut microbiomes of carbapenemase-producing Enterobacteriaceae colonized subjects

**DOI:** 10.1038/s41564-022-01221-w

**Published:** 2022-09-15

**Authors:** Jonathan T. L. Kang, Jonathan J. Y. Teo, Denis Bertrand, Amanda Ng, Aarthi Ravikrishnan, Melvin Yong, Oon Tek Ng, Kalisvar Marimuthu, Swaine L. Chen, Kern Rei Chng, Yunn-Hwen Gan, Niranjan Nagarajan

**Affiliations:** 1 Genome Institute of Singapore, Singapore, Singapore; 2 Yong Loo Lin School of Medicine, National University of Singapore, Singapore, Singapore; 3 Institute of Infectious Diseases and Epidemiology, Tan Tock Seng Hospital, Singapore, Singapore

**Keywords:** Antimicrobial resistance, Microbiome, Microbial genetics, Microbial ecology, Genome informatics

## Abstract

Long-term colonization of the gut microbiome by carbapenemase-producing Enterobacteriaceae (CPE) is a growing area of public health concern as it can lead to community transmission and rapid increase in cases of life-threatening CPE infections. Here, leveraging the observation that many subjects are decolonized without interventions within a year, we used longitudinal shotgun metagenomics (up to 12 timepoints) for detailed characterization of ecological and evolutionary dynamics in the gut microbiome of a cohort of CPE-colonized subjects and family members (*n* = 46; 361 samples). Subjects who underwent decolonization exhibited a distinct ecological shift marked by recovery of microbial diversity, key commensals and anti-inflammatory pathways. In addition, colonization was marked by elevated but unstable Enterobacteriaceae abundances, which exhibited distinct strain-level dynamics for different species (*Escherichia coli* and *Klebsiella pneumoniae*). Finally, comparative analysis with whole-genome sequencing data from CPE isolates (*n* = 159) helped identify substrain variation in key functional genes and the presence of highly similar *E. coli* and *K. pneumoniae* strains with variable resistance profiles and plasmid sharing. These results provide an enhanced view into how colonization by multi-drug-resistant bacteria associates with altered gut ecology and can enable transfer of resistance genes, even in the absence of overt infection and antibiotic usage.

## Abstract (web summary)

Longitudinal shotgun metagenomics reveal changes in the gut microbial ecology upon carbapenemase-producing Enterobacteriaceae colonization and decolonization of adult subjects.

## Main

The global dissemination of antibiotic resistance genes (ARGs) among pathogenic bacteria is a major public health problem that, if left unaddressed, would reduce efficacy of treatments, elevate costs and increase mortality^1^. Of particular concern is the spread of carbapenemase-producing Enterobacteriaceae (CPE)^2^, with their ability to degrade carbapenems often acquired from plasmids encoding carbapenemases^3^, thus rapidly endangering these antibiotics of last resort^4^. In addition to life-threatening infections, asymptomatic gut colonization by CPE is increasingly common^5^, creating ARG transmission reservoirs^6^. While previous studies have focused on epidemiology^2,7^ and molecular aspects^4,8^, the natural history of gut colonization including ecological and evolutionary changes linked to ARG transmission or CPE decolonization remains unexplored.

Recent studies into host–microbiome–pathogen interactions have provided important insights into pathogenesis^9^, immune response^10^ and treatment avenues^11^ for various pathogens. Some have leveraged metagenomics to temporally track microbiomes and understand ecological responses to overt infection^11^. As microbes have rapid turnover, whole-genome sequencing (WGS) of pathogens has helped characterize intra-host evolution during chronic infections, identifying key enzymes for host adaptation or colonization^12^. Alternatively, deep metagenomic sequencing can reveal nucleotide-level variation for many species of interest^13^, shedding light on strain-level dynamics in microbiomes. This approach has characterized stable microbiomes in healthy individuals as well as dynamic changes during faecal microbiota transplantation^14^. Asymptomatic gut colonization by CPE strains presents a unique opportunity to study an intermediate phenomenon, that is, strain competition with commensals, and associated ecological and evolutionary adaptations, without overt infection or disease.

In this Letter, we conducted longitudinal gut microbial analysis for a cohort of index subjects (*n* = 29, CPE colonized at recruitment) and their family members (*n* = 17, not CPE colonized) with up to 12 timepoints within a year, to obtain multi-scale^15^ (microbiome composition-, strain- and gene-level) characterization of ecological and evolutionary changes during CPE colonization. On the basis of deep shotgun metagenomics of stool DNA, we observed distinct ecological shifts marked by recovery of diversity and key commensals in association with CPE decolonization. CPE colonization was marked by elevated but unstable Enterobacteriaceae abundances, which exhibited specific strain-level dynamics for different species (*Escherichia coli* and *Klebsiella pneumoniae*). Comparative analysis with WGS data from CPE isolates (*n* = 159) identified the presence of highly similar *E. coli* and *K. pneumoniae* strains with variable resistance profiles and plasmid sharing. These results provide an enhanced view into how colonization by multi-drug-resistant bacteria associates with altered gut ecology and can enable transfer of resistance genes, even without overt infection.

## Results

### Ecological shifts and recovery after CPE decolonization

**Fig. 1:**
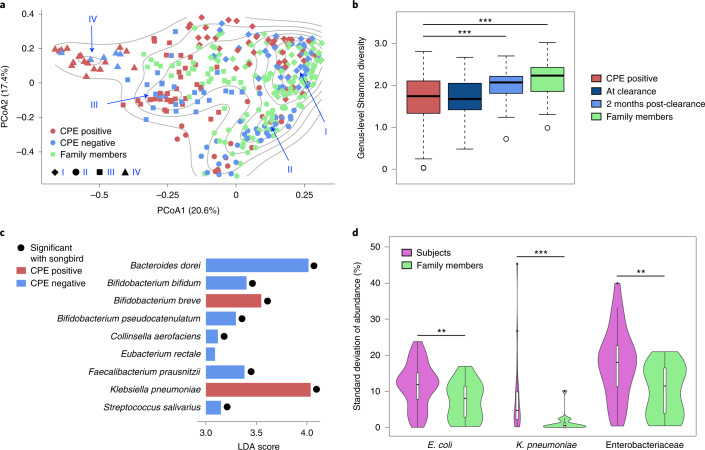
Shifts in gut microbial ecology associated with CPE colonization. **a**, PCoA plot showing how gut microbial community composition varies in relation to CPE colonization status (genus-level Bray–Curtis dissimilarity; Unifrac plot in Extended Data Fig. 1). Contour lines indicate similar density with regions of locally higher density associated with distinct community configurations (defined by average linkage clustering in Extended Data Fig. 1; marked with labels I, II, III and IV). **b**, Box plots showing genus-level Shannon diversity distributions for different timepoints for index patients (‘CPE positive’, during colonization; ‘At clearance’, within 1 month of decolonization; ‘2 months post-clearance’, timepoints that were >2 months after decolonization) and all timepoints for family members (*n* = 347 timepoints; two-sided Wilcoxon rank-sum test *P* values: CPE positive versus 2 months post-clearance, 1.15 × 10^−4^; CPE positive versus family members, 2.46 × 10^−12^). **c**, Species that were found to be enriched in CPE-positive (red) and CPE-negative (blue) samples along with their linear discriminant analysis (LDA) scores based on LEfSe analysis. Results that were significant on the basis of Songbird analysis as well are indicated with a solid circle. **d**, Violin plots showing the standard deviation of relative abundances (ignoring relative abundances <0.1% to avoid the effect of detection limit for metagenomics) over time of various taxa in different individuals (subjects and family members) (*n* = 43 individuals, two-sided Wilcoxon rank-sum test *P* values: *E. coli*, 0.021; *K. pneumoniae*, 4.96 × 10^−5^; Enterobacteriaceae, 0.011). For all subfigures: *P < 0.1, **P < 0.05, ***P < 0.01 based on the Wilcoxon rank-sum test, and all other comparisons were not statistically significant. Centre lines in the boxplots represent median values, box limits represent upper and lower quartile values, and whiskers represent 1.5 times the interquartile range above the upper quartile and below the lower quartile. Source data

**Extended Data Fig. 1:**
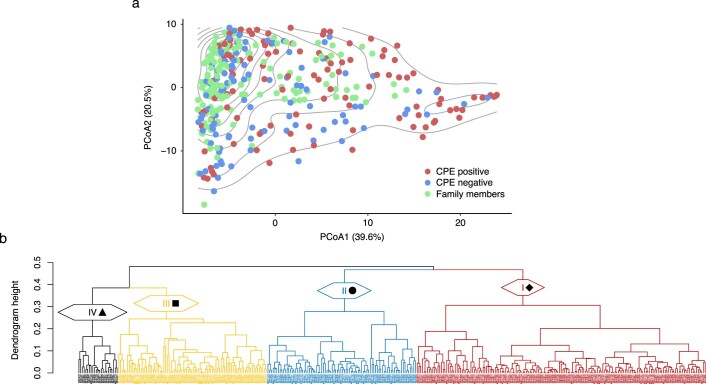
Additional principal coordinates analysis. (**a**) Principal coordinates analysis (PCoA) plot similar to Fig. 1a but calculated based on genus-level weighted UniFrac distance. Note that while the location of points is flipped with respect to the x-axis for Fig. 1a, the general distribution of points is similar with regions of high density on the left having more family members while a less dense cluster on the right has more CPE positive points. (**b**) Dendrogram for hierarchical clustering based on distance in PCoA1-PCoA2 space in Fig. 1a, and number of clusters determined by AIC. The labels I–IV correspond to those in Fig. 1a. Dendrogram tips are labeled with the library ID of each sample (see Supplementary File 1). Source data

**Extended Data Fig. 2:**
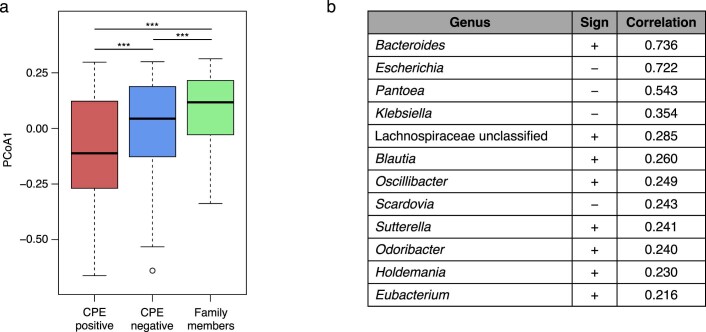
Associations with the first principal coordinate. (**a**) Comparison of principal coordinates analysis (PCoA) values (Fig. 1a) across different groups of samples based on the first principal coordinate (PCoA1). Wilcoxon rank-sum p < 0.01 for all pairwise comparisons (n = 362 samples; two-sided Wilcoxon rank-sum test p-values – CPE positive vs. CPE negative: 5.14 × 10^−4^, CPE negative vs. family members: 0.00694, CPE positive vs. family members: 7.48 × 10^−11^). Centre lines in the boxplots represent median values, box limits represent upper and lower quartile values, and whiskers represent 1.5 times the interquartile range above the upper quartile and below the lower quartile. (**b**) Genera most correlated with PCoA1 (Fig. 1a). Sign (−): Negative correlation with PCoA1 (most abundant in configuration IV samples). Sign (+): Positive correlation with PCoA1 (least abundant in configuration IV samples). Source data

Leveraging the observation that CPE carriage is resolved within 3 months, with 98.5% probability within a year for our cohort^16^ (longer for other cohorts^17^), we tracked gut microbiome composition in these individuals for a year to understand ecological changes associated with decolonization (12 timepoints, 361 samples in total; Extended Data Table 1 and Supplementary File 1). Specifically, stool samples were obtained from hospital patients who screened positive for CPE carriage (*n* = 29, index subjects) and non-CPE-colonized family members (*n* = 17, home-environment-matched controls) and characterized via deep shotgun metagenomics (>50 million 2 × 100 bp reads; Methods). Principal coordinates analysis (PCoA) with average-linkage clustering based on taxonomic profiles identified multiple distinct community configurations (I, II, III and IV), where CPE-positive samples (from stool culture and quantitative PCR^16^) were less common in configurations I and II, and more common in configurations III and IV (Fig. 1a, Extended Data Fig. 1 and Supplementary File 2). This statistically significant shift of CPE-positive samples along PCoA1 (Wilcoxon *P* value < 1.4 × 10^−8^; Extended Data Fig. 2a) is defined by a gradient of abundances strongly correlated with *Escherichia* (negative, that is, more abundant in configuration IV samples) and *Bacteroides* (positive; Extended Data Fig. 2b). A similar shift was observed when comparing taxonomic profiles for configuration IV versus configuration I (Supplementary File 3). Interestingly, while configuration IV has no microbiomes from family members, a few CPE-negative samples from index subjects also cluster here.

Grouping samples on the basis of temporal proximity to CPE clearance highlighted that, while CPE-positive samples have lowest average diversity^18^, there is a gradual increase around the time of decolonization and post-decolonization, with diversity reaching the higher levels seen in family members after 2 months (Fig. 1b). This pattern was seen even after accounting for potential confounders, including antibiotic usage, hospitalization status, multiple timepoints for an individual, gender and ethnicity, in a linear mixed-effects model (Supplementary Fig. 1 and Methods). We investigated whether CPE colonization alone could explain these changes by computationally subtracting Enterobacteriaceae from taxonomic profiles and recomputing diversity metrics. We noted that richness and Shannon diversity consistently preserved the trend of increasing during decolonization (Supplementary Fig. 2), pointing to a wider microbiome shift besides Enterobacteriaceae.

**Extended Data Fig. 3:**
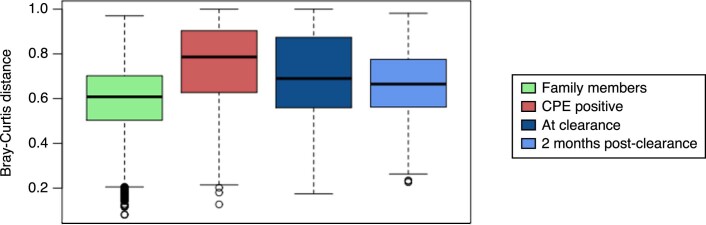
Pairwise genus-level Bray-Curtis distances with family members. Boxplots quantifying the distributions of pairwise genus-level Bray-Curtis distances. Each pair consists of one sample from a family member, and a second sample from one of the following groups: family members, CPE positive, CPE negative at point of clearance, as well as more than two months post-clearance. Wilcoxon rank-sum p < 0.01 for all pairwise comparisons with the family member group. (n = 39,420 sample pairs; two-sided Wilcoxon rank-sum test p-values of comparison with the family member group – CPE positive: <2.2 × 10^−16^; At clearance: <2.2 × 10^−16^; 2 months post-clearance: <2.2 × 10^−16^). Centre lines in the boxplots represent median values, box limits represent upper and lower quartile values, and whiskers represent 1.5 times the interquartile range above the upper quartile and below the lower quartile. Source data

Shifts in diversity during decolonization were also reflected in microbiome similarity, with Bray–Curtis distances to family members being highest in CPE-positive samples, reducing during and post-decolonization towards baseline values seen among family members (Extended Data Fig. 3). These results highlight the ecological shift associated with CPE colonization that resolves post-decolonization, but with residual effects in some individuals.

To identify species associated with decolonization, we conducted differential-abundance analysis based on CPE status (Methods and Supplementary File 2). While most Enterobacteriaceae species were not differentially abundant, *K. pneumoniae*^2,4^ had strong association with CPE-positive status (Fig. 1c). Only one other species (*Bifidobacterium breve*) was enriched in CPE-positive samples, while seven species were depleted in CPE-negative samples. These included important commensals known to reduce gut inflammation through diverse pathways, including *Bacteroides dorei* (decreasing gut microbial lipopolysaccharide production^19^), *Faecalibacterium prausnitzii* (butyrate production^20^) and *Bifidobacterium* (*bifidum*, *pseudocatenulatum*; inhibition of NF-κB activation^21^), and could thus aid CPE decolonization^22^.

**Extended Data Fig. 4:**
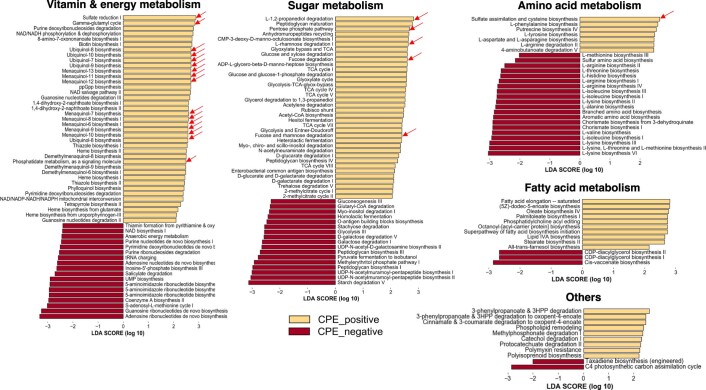
Pathway comparisons in colonized and decolonized gut metagenomes. Differentially abundant pathways in colonized (CPE positive) and post-decolonization (CPE negative) gut metagenomes (two-sided Wilcoxon rank-sum test FDR-adjusted p-value<0.05, LDA score>2). Pathways highlighted with an arrow are those that have potential associations with gut inflammation and aerobic respiration. Source data

Differential pathway analysis based on CPE status provided further supporting evidence that key inflammatory pathways (for example, sulphate reduction) are enriched during colonization, highlighting their role in the process (Extended Data Fig. 4 and Supplementary File 4). Furthermore, aerobic respiration and oxidative phosphorylation pathways (for example, pentose phosphate) were enriched during CPE colonization consistent with a model of gut oxygenation proposed previously^23^. These results were recapitulated after excluding Enterobacteriaceae from functional profiles, emphasizing that they are not solely from CPE colonization and have substantial contributions from other species (Supplementary Fig. 3). Micro-aerophilic niches for Enterobacteriaceae due to antibiotic treatment provide another potential explanation^24^, as antibiotic usage was common (preceding ~25% timepoints; Supplementary File 1). Notably, while ARGs were enriched in gut microbiomes for CPE-positive timepoints, no significant differences were observed between index subjects and family members at other timepoints (Supplementary Fig. 4).

**Extended Data Fig. 5:**
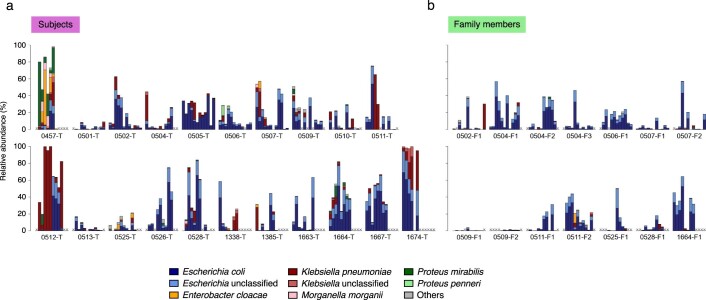
Enterobacteriaceae relative abundances. Bar plots showing the relative abundances of various Enterobacteriaceae species in (**a**) subjects and (**b**) family members, over the course of sample collection. Each sub-panel consists of 12 bars, representing consecutively visit 0 (V00) to visit 11 (V11). An ‘X’ at a particular position indicates that a sample corresponding to that visit number was not collected. Only individuals with samples collected at six or more visits are shown. Source data

While Enterobacteriaceae species were enriched in CPE-positive samples relative to CPE-negative samples (Wilcoxon *P* value < 3.5 × 10^−5^), index subjects at CPE-negative timepoints also showed enrichment compared with family members (Wilcoxon *P* value = 0.01; Extended Data Fig. 5). Overall, Enterobacteriaceae composition varied across individuals, with *E. coli* and *K. pneumoniae* being most common, but other *Escherichia*, *Klebsiella*, *Enterobacter* and *Proteus* species also being moderately abundant across individuals and timepoints (Extended Data Fig. 5). The abundant Enterobacteriaceae species in an individual were not necessarily the CPE species (for example, subject 0505-T, timepoints 1–3). Enterobacteriaceae profiles also shifted rapidly (for example, in 0457-T and 0512-T, timepoint 6) with higher temporal variation in index subjects (Wilcoxon *P* value < 0.05; Fig. 1d and Extended Data Fig. 5). Together, these results suggest that CPE colonization is maintained by an altered, dynamic pro-inflammatory microenvironment supporting Enterobacteriaceae, which resolves concurrently with microbiome recovery^25^.

### Distinct strain-level dynamics of Enterobacteriaceae species

**Fig. 2:**
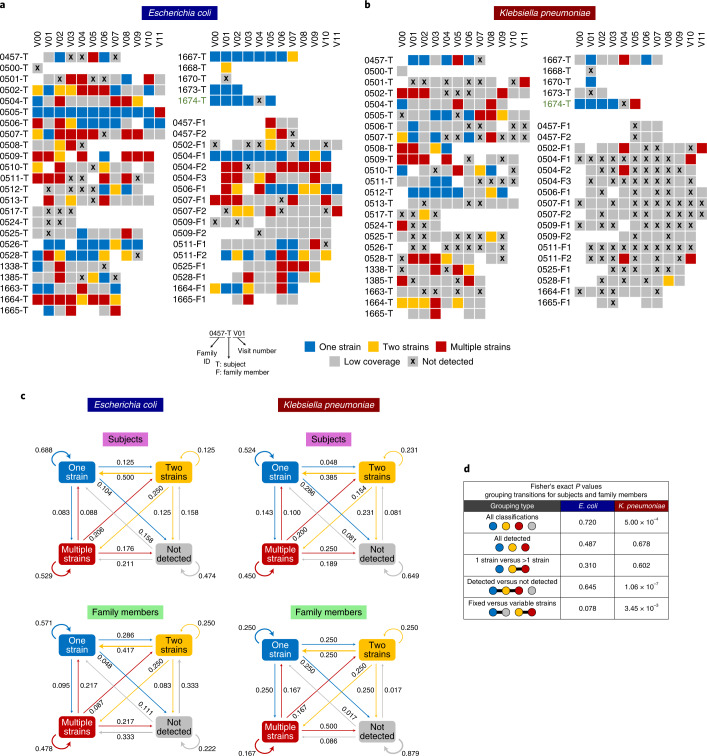
*Enterobacteriaceae* strain variations and dynamics in index subjects and family members. **a**,**b**, Strain composition for *E. coli* (**a**) and *K. pneumoniae* (**b**) determined on the basis of allele frequency distributions per sample. Samples where a species was not detected (relative abundance <0.1%) or in which the genome had low coverage (<10×) were distinguished from those where one, two or more strains were confidently detected. Each row depicts the multiple timepoints for a subject (where available), with index subjects indicated with a T and family members with an F in subject IDs. **c**, First-order Markov models showing the probability of transition between different states (excluding the low coverage state where information is missing). **d**, Table showing statistical significance of differences in transition probabilities between index subjects and family members, for various groupings of states and Enterobacteriaceae species. States placed in the same grouping are connected by a horizontal line. Source data

Analysing the deep metagenomic data at higher resolution, we investigated strain-level dynamics for the two most prevalent Enterobacteriaceae (*E. coli* and *K. pneumoniae*). Read mapping to references identified single-nucleotide variants (SNVs), and allele frequency modes were used with a classical population genetics approach to determine strain count^13^ (Methods and Supplementary Fig. 5). For 53% of samples (*E. coli*, 63%; *K. pneumoniae*, 38%) where a species was detected (abundance >0.1%), read coverage was sufficient to identify strain count (one, two or multiple strains, otherwise low coverage; Fig. 2a,b and Supplementary Fig. 6). As expected for a gut commensal^26^, *E. coli* was at comparable frequencies in index subjects (86%) and family members (90%), and more frequently detected relative to *K. pneumoniae* (Fisher’s exact *P* value < 5 × 10^−20^; Fig. 2a). *K. pneumoniae* was, however, more frequently found in index subjects (70%) relative to family members (39%), consistent with the hypothesis that a pro-inflammatory environment might be facilitating colonization (Fisher’s exact *P* value < 2 × 10^−9^; Fig. 2b).

Among classified samples, *E. coli* was frequently single strain in index patients (44%) while family members often had multiple strains (44%; Supplementary Fig. 6), pointing to clonal dominance in pro-inflammatory environments. A few individuals maintained a single strain over several months (for example, 0505-T, 1667-T and 0506-T), indicating that this can be a stable state (Fig. 2a). For *K. pneumoniae* despite sporadic detection, the multi-strain state was more common (49%), consistent with the hypothesis that even in pro-inflammatory environments, no clone outcompetes others^27^ (Fig. 2b and Supplementary Fig. 6). Overall, in agreement with previous observations (Fig. 1d and Extended Data Fig. 5), Enterobacteriaceae strain compositions were highly variable temporally.

Capturing transition frequencies between strain compositions as first-order Markov models, we noted distinct patterns for *E. coli* and *K. pneumoniae*, and index subjects versus family members (Fig. 2c and Methods). For example, *E. coli* colonization is more likely to stay single strain for index patients (69%) versus family members (57%) and single-strain *K. pneumoniae* colonization (52%; Fig. 2c). When *E. coli* is not detected, this state is more likely to be maintained in index subjects (47%) versus family members (22%; Fig. 2c). Overall, the Markov model predicts that *E. coli* is maintained as single strains (index subjects, 41%; family members, 39%). Contrastingly, *K. pneumoniae* frequently converges to not detected state in subjects (42%) and family members (75%). Grouping classes of strain detection, we tested if transition probabilities differed across cohorts and species (Fig. 2d). For *E. coli*, transition probabilities were not significantly different between cohorts (Fisher’s exact *P* value > 0.05; Fig. 2d). In contrast, driven by stark detected/not detected patterns for *K. pneumoniae*, index subjects had significantly different transition probabilities compared with family members for various groupings involving the not detected state (‘All classifications’, ‘Detected versus not detected’ and ‘Fixed versus variable strains’; Fisher’s exact *P* value < 10^−2^; Fig. 2d). These results further highlight differences in strain-level dynamics for Enterobacteriaceae in the potentially pro-inflammatory gut microbiome milieu of index subjects.

### Substrain variation and plasmid sharing in Enterobacteriaceae

**Extended Data Fig. 6:**
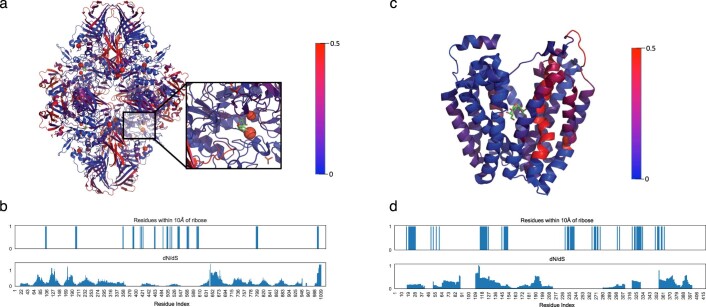
dN/dS values in *lacZ* and *lacY*. (**a**) Visualization of dN/dS rates for the *lacZ* tetramer (4dux) using a 30aa window based on metagenomics-derived SNVs (Table 1). The inset shows a close-up view of *lacZ*’s sugar binding active site. In general, dN/dS rates were lower for *lacZ* in regions close to the active site (two-sided Wilcoxon rank-sum test p-value<2.6 × 10^−9^) except for around position 1000. (**b**) Plot of dN/dS values and regions of *lacZ* that are close to ribose. (**c**) Visualization of dN/dS rates for *lacY* (1pv7) using a 30aa window. Interestingly, *lacY* exhibited a distinct trend with a few regions of higher dN/dS rates close to the active site (two-sided Wilcoxon rank-sum test<0.029). (**d**) Plot of dN/dS values and regions close to the *lacY* lactose symporter channel. Source data

Samples determined to have a single strain can still exhibit substrain variation relative to this genomic background, similar to viral quasi-species. Characterizing distribution of such intra-host variations across genes could identify adaptive changes important for CPE colonization, similar to recent studies with mouse models and strain isolates^28,29^. To analyse standing variation in Enterobacteriaceae, we identified low-frequency (<50%) SNVs in single-strain timepoints (*E. coli*, 30,155; *K. pneumoniae*, 13,061), annotating them for function-altering changes to reveal adaptive signatures in Enterobacteriaceae during gut colonization (Supplementary File 5 and Methods). We found 5,919 and 1,787 function-altering changes in *E. coli* and *K. pneumoniae*, including several in key polysaccharide utilization and virulence genes (for example, *lacZ, lacY* and ECIAI39_4258 (putative invasin/intimin protein)) similar to key genes previously identified from isolate WGS as undergoing selection for human gut colonization^30^ (Table 1). Visualizing function-altering SNVs in polysaccharide-utilization genes, where adaptive mutations can reflect pressures to use diet-derived polysaccharides, identified structural motifs that might be key functionally (Extended Data Fig. 6). Consistent with our study of low-frequency SNVs, most regions bear signatures of purifying selection (dN/dS < 0.5; Extended Data Fig. 6), though identified genes were significantly enriched relative to genome-wide average for non-synonymous SNVs (Table 1).

**Table 1:** Top genes with substrain variation

Gene	Protein	SNV count	*P* value
*E. coli*			
**ECIAI39_4258**	Putative invasin/intimin protein	48	<10^−^^10^
**ECIAI39_0530**	Host specificity protein J of prophage	34	<10^−13^
***ydcM***	Putative transposase	24	<10^−15^
**ECIAI39_1027**	Putative GTP-binding domain	19	<10^−12^
**ECIAI39_4240**	Putative anti-restriction protein	18	<10^−14^
***yeeS***	Putative DNA repair protein; CP4-44 prophage	18	<10^−16^
***lacZ***	β-d-galactosidase	17	<0.05
**ECIAI39_4893**	Putative tail fibre component K of prophage	14	<10^−7^
***lacY***	Lactose/galactose transporter	14	<10^−4^
***Rz***	Endopeptidase from phage origin (lysis protein)	13	<10^−9^
**ECIAI39_1018**	Hypothetical protein	11	<10^−4^
**ECIAI39_4867**	Hypothetical protein	10	<10^−2^
***yncK***	Transposase ORF A	10	<10^−8^

*K. pneumoniae*			
**KPHS_51490**	Putative transposase	30	<10^−39^
**KPHS_35720**	Putative transposase	18	<10^−13^
**KPHS_22580**	Hypothetical protein	12	<10^−4^

Top ten genes with most function-altering SNVs (≥10) with low frequencies (allele frequency < 0.5) for *E. coli* and *K. pneumoniae*. SNVs in distinct individuals were counted separately, but multiple timepoints were counted as one. *P* values were computed by performing a binomial test on the observed number of SNVs given the probability of a gene acquiring SNVs after accounting for (1) the probabilities of acquiring mutations across all genes and (2) the codon composition of the genes where the respective SNVs are found. Bonferroni correction (α = 0.05) was applied.

**Fig. 3:**
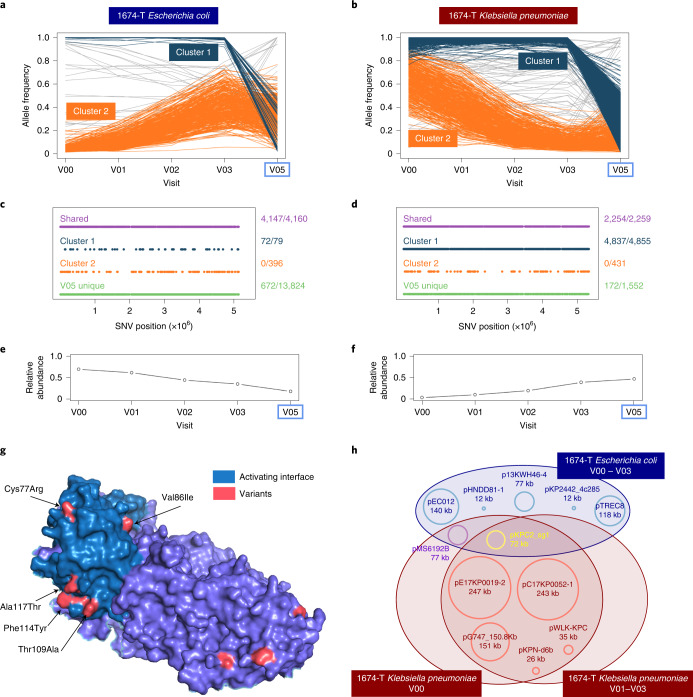
Substrain variation and plasmid sharing in Enterobacteriaceae species. **a**,**b**, Line charts showing the allele frequencies of metagenomics-derived SNVs across timepoints for *E. coli* (**a**) and *K. pneumoniae* (**b**), for subject 1674-T. SNVs that belong to different substrain clusters are coloured accordingly, and a potential model for the corresponding haplotypes is shown in Supplementary Fig. 7. All genome-wide SNVs detected at all the depicted timepoints are shown here. The blue box indicates the CPE-negative timepoint. **c**,**d**, Corresponding genomic distribution for SNVs belonging to cluster 1 or cluster 2 and those that are fixed at all timepoints (‘Shared’) or just at the CPE-negative timepoint (‘V05 unique’). The associated fraction describes the number of SNVs in a grouping that are common with CPE isolates, in relation to the total number of SNVs in this grouping. **e**,**f**, Relative abundance of *E. coli* and *K. pneumoniae* in the gut metagenome for subject 1674-T across timepoints. **g**, Visualization of amino acid changes (red) seen in a key polysaccharide utilization protein (lacZ, pdb structure 4DUX chain A, identified from Table 1 and Extended Data Table 2) based on metagenomics-derived SNVs that changed in frequency over time and across multiple individuals. SNVs are found primarily on the protein surface, with six SNVs located on the activating interface responsible for tetramerization (teal). **h**, Diagram depicting plasmid sharing between *E. coli* and *K. pneumonia*e strains at various timepoints for subject 1674-T. Plasmid sequences were clustered at 95% similarity (Supplementary Fig. 8), and a representative plasmid for each cluster is shown in the Venn diagram as an approximately sized circle, with plasmid name and size. Source data

**Extended Data Fig. 7:**
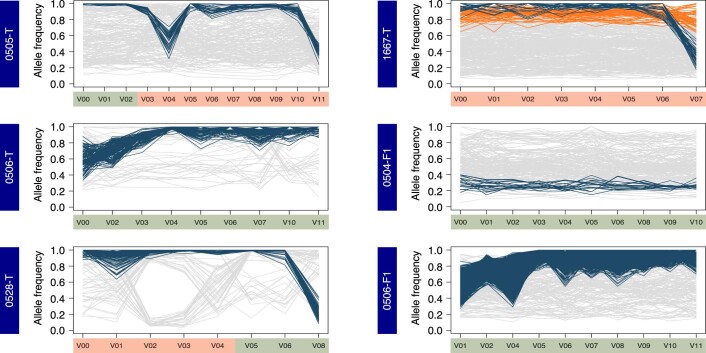
Sub-strain variations in *E. coli*. Sub-strain variations over time for other individuals (*E. coli*). Selected figures were plotted for subjects with >3 consecutive single-strain timepoints. Coloured lines depict clusters of SNVs that were identified. Timepoints are shaded based on whether they were determined to be CPE colonized (red) or not (green). Note that while a cluster of SNVs typically tends to be dominant in a subject across timepoints (coloured in dark blue, except for 0504-F1), they can drop in frequency at some timepoints, and this does not necessarily coincide with the CPE decolonization event as was observed for 1674-T (for example, V08 for 0528-T). Source data

**Extended Data Fig. 8:**
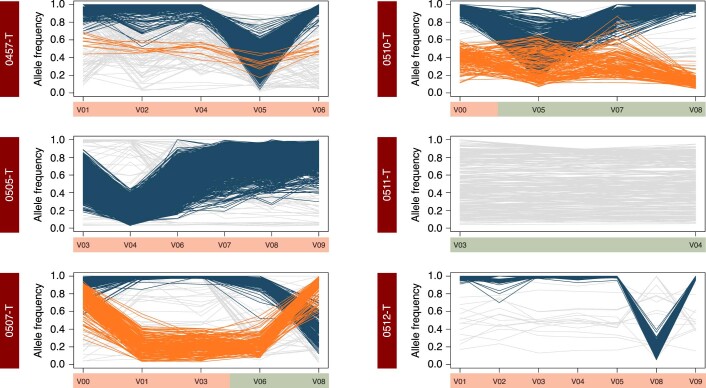
Sub-strain variations in *K. pneumoniae*. Sub-strain variations over time for other individuals (*K. pneumoniae*). Figures were plotted for subjects with ≥2 single-strain timepoints. Coloured lines depict clusters of SNVs that were identified. Timepoints are shaded based on whether they were determined to be CPE colonized (red) or not (green). Note that similar to the observation in Extended Data Fig. 7, the dominant sub-strain in a subject can switch at timepoints that do not coincide with change in CPE colonization status (for example, V01 and V08 for 0507-T). Source data

To study SNVs further relative to CPE decolonization, time-series information was used to cluster co-varying SNVs (Methods). Interestingly, in some subjects multiple clusters were identified, revealing distinct substrain lineages differing by few hundred SNVs genome-wide (for example, 1674-T; Fig. 3a,b, Extended Data Figs. 7 and 8 and Supplementary Fig. 7). For subject 1674-T, both *E. coli* and *K. pneumoniae* have a dominant cluster during CPE-positive timepoints (V00–V03) that match SNV signatures seen in corresponding CPE isolates (*Shared* and *Cluster 1* SNVs; Fig. 3c,d and Supplementary Fig. 7). The subdominant cluster (*Cluster 2*; Fig. 3c,d and Supplementary Fig. 7; possibly sublineage of *Cluster 1*) has SNV signature not seen in CPE isolates but detected post-decolonization (V05), indicating that these lineages are discordant for CPE status despite high genomic similarity. For both species, decolonization coincides with appearance of distinct strains with >1,000 SNVs relative to CPE strains (*V05 unique*; Fig. 3c,d and Supplementary Fig. 7). Despite shared strain patterns for *E. coli* and *K. pneumoniae*, they exhibited dissimilar relative abundance trends, with *E. coli* being reduced at decolonization (V05) while *K. pneumoniae* peaks at this point (Fig. 3e,f). While a few dense trajectories of co-varying SNVs were detected in other individuals, many SNVs varied independently of these clusters (Extended Data Figs. 7 and 8). These results suggest that Enterobacteriaceae may share substrain dynamics during CPE decolonization while having species-specific ecological properties.

Leveraging availability of multiple timepoints across subjects, we identified SNVs whose frequencies varied notably over time (>30%). These were overlapped across subjects to identify SNVs that recurrently vary (Extended Data Table 2 and Supplementary File 6), identifying several polysaccharide utilization (*lacZ, lacI* and *treA*), pyruvate metabolism (*pflB* and *pykF*) and protein synthesis (*dnaK*, 30S and 50S ribosomal subunits) genes implicated in adaptation to nutrient limitation^31^, antibiotics^32^ and environmental stress^33^ conditions. Several genes were common to *E. coli* and *K. pneumoniae* (*srmB*, *pnp*, *nlpI* and *pheT*), indicating that similar selection constraints may act on strains for both species. The *lacZ* gene was highlighted as having the most recurrent, frequency-varying SNVs (*n* = 12), with all SNVs occurring in surface-exposed regions (Fig. 3g). Comparing accessible surface area of variant residues versus others highlighted that variants are significantly more exposed to solvent (mean 73.1Å^2^ versus 34.1Å^2^, Welch’s *t*-test *P* value < 0.01). Many SNVs occurred in the activating interface, a region near amino-terminus required for tetramerization^34^, indicating that they could impact lacZ function via complex formation dynamics.

Among genetic features prominent in CPE strains from subject 1674-T, variants in polysaccharide utilization genes were common as discussed previously (Fig. 3g). Additionally, plasmid sequence analysis identified two shared plasmids between *E. coli* and *K. pneumoniae* CPE isolates (Fig. 3h, Supplementary Fig. 8 and Methods). This included pKPC2, recently identified in hypervirulent carbapenem-resistant *K. pneumoniae* from Singapore, and harbouring *bla*_KPC-2_, the carbapenemase that provided CPE designation for these isolates^16^. Additionally, pMS6192B was shared between all *E. coli* and *K. pneumoniae* from the first visit (V00; Fig. 3h). The shared plasmids have total sequence length >140 kbp and no SNVs across species, indicating a recent common source. Plasmid transfer experiments with pKPC2 between *E. coli* and *K. pneumoniae* suggest moderate conjugation frequency under in vitro conditions (~0.1%; Methods). Furthermore, 50% of plasmid-bearing clones (3/6) were observed to have SNV in pKPC2 after 300 generations, defining an upper-bound on divergence of plasmid-bearing isolates having no SNVs being 5 months (binomial *P* value < 0.05).

## Discussion

The availability of metagenomic data for up to 12 timepoints over a year in this study allowed examination of long-term dynamics, enabling microbiome comparisons before and after CPE decolonization in a subject-matched fashion to study microbiome shifts associated with decolonization. This revealed ecological shifts that cannot be explained solely by CPE strain loss (for example, increase in species richness), and the specific taxonomic/functional changes observed point to the role of inflammation in maintaining Enterobacteriaceae-favourable gut environment in index subjects (for example, *Pantoea* species; Extended Data Fig. 2 and Supplementary File 3). Additionally, our data indicate that this configuration may be unstable in many individuals, opening up the possibility that interventions that reduce gut inflammation directly or via action of probiotics could reduce Enterobacteriaceae and promote CPE decolonization.

Gut inflammation has been known to create a niche for enterics such as *Salmonella*^35^, where some species use sulphate, nitrate and tetrathionate as terminal electron acceptors for anaerobic respiration (for example, *E. coli*). The enriched pathways in CPE-colonized subjects are marked by menaquinol biosynthesis, glycolysis and respiration (tricarboxylic acid cycle), even after computationally subtracting Enterobacteriaceae contributions, indicating that the gut environment in this group is qualitatively different in oxygenation. Additionally, fucose/rhamnose and 1,2-propanediol degradation are enriched during CPE colonization, potentially serving as carbon sources for Enterobacteriaceae such as *K. pneumoniae*, which demonstrates competitive fitness with oxygen as terminal electron acceptor under such conditions^27^. The enrichment of pentose phosphate pathway could indicate need for reducing equivalents of NADPH^+^ to maintain redox conditions or serve as nucleic acid precursors to fuel growth. Overall, the shift in microbial pathways during CPE colonization is independent of Enterobacteriaceae but favours their growth. Further work is needed to understand if this shift is established by gut inflammation (as in colitis^36^, via direct biomarker measurement, for example, calprotectin) or if diverse factors play a strong role in an individual-specific manner (for example, antibiotics for some subjects^24^). Notably, while reduction in microbial diversity during CPE colonization could not be attributed solely to antibiotic usage or hospitalization at a timepoint, these could be delayed effects and would need a more controlled study design to explore further.

An alternative strategy for CPE decolonization involves introduction of species depleted in the colonized state (for example, *Faecalibacterium prausnitzii* or *Bifidobacterium bifidum*), either as probiotic formulations or through faecal microbiota transplants^37^. Matching donors to recipients, supplementing missing species or promoting further instability in Enterobacteriaceae abundances based on ecological models could be a promising avenue to explore, similar to studies for *Clostridoides difficile*^38^. The observed differences in colonization dynamics for Enterobacteriaceae suggest that decolonization strategies might also have to be species specific. For example, a possible explanation for presence of multiple *K. pneumoniae* strains is that they are not well adapted for gut colonization but instead opportunistically exploit a niche. Decolonization of *K. pneumoniae* strains may therefore require elimination of conditions favouring this niche such as inflammation or availability of specific sugars. The presence of single *E. coli* strains in many subjects supports a model where gut-adapted strains acquire ARGs, and plasmid-targeting strategies might be better suited in this case. Interestingly, our data suggest that human gut microbiomes can harbour multiple strains of commensals such as *E. coli* (in contrast to observations in mouse studies^39^), even among non-CPE-colonized family members^40^ (Fig. 2a). Further studies using high-throughput culturing and single-cell sequencing could help reconstruct strain genomes and unravel factors that determine niche competition^41^.

Identifying genes that enable CPE gut colonization provides another avenue to target interventions. As shown here, analysis of high-coverage metagenomic data to identify substrain variations can provide promising hypotheses based on in vivo evolution, similar to viral quasi-species analysis^42^, or mutagenesis-based experiments^43^. Furthermore, isolation of lineages with distinct advantages in colonizing or avoiding decolonization (for example, cluster 2 in 1674-T), could narrow down genetic features to be investigated in vitro. Finally, the role of gut microbiome as an ARG reservoir, and plasmid sharing across Enterobacteriaceae, is of particular concern. While we cannot definitively conclude that data for 1674-T represent plasmid transfer, these observations and isolated strains serve as important resources for further investigations into plasmid transmission and CPE decolonization.

## Methods

### Sample collection and CPE classification

A prospective cohort study involving CPE carriers was conducted from October 2016 to February 2018. Study participants were recruited with informed consent from two tertiary healthcare centres in Singapore. CPE carriers were identified by routine collection of rectal swab samples for clinical care and/or infection prevention and control measures, in accordance with local infection control policies. The study received ethics approval from the Singapore National Healthcare Group Domain Specific Review Board 74 (NHG DSRB Reference 2016/00364) before commencement. Stool samples were first collected weekly for 4 weeks, then monthly for 5 months and finally once every 2 months for 6 months. In addition to the CPE-colonized subjects, stool samples from a number of family members were also obtained to provide a control dataset. Samples obtained from index subjects were classified as either CPE positive or CPE negative, on the basis of whether CPE genes (including *bla*_NDM-1_, *bla*_KPC_, *bla*_OXA-48_, *bla*_IMI-1_ and *bla*_IMP_) were positively identified from Enterobacteriaceae isolates found to be resistant to either meropenem or ertapenem^16^ (Supplementary Table 1). The presence of CPE-negative samples was used to detect CPE clearance, and samples were further classified on the basis of the amount of time elapsed since clearance, that is, before clearance, within 2 months post-clearance and more than 2 months post-clearance. Owing to the focus on household transmission and CPE carriage, dietary information was not collected in the study.

### Isolate sequencing and assembly

DNA for all CPE isolates obtained from stool samples in this study (all subjects, all timepoints) was collected from Tan Tock Seng Hospital and transferred to the Genome Institute of Singapore (GIS) for WGS. Library preparation was performed using the NEBNext Ultra DNA Library Prep Kit for Illumina, and 2 × 151 base-pair sequencing was performed using the Illumina HiSeq 4000. Raw FASTQ reads were processed using in-house pipelines at GIS for de novo assembly with the Velvet assembler^44^ (v1.2.10), parameters optimized by Velvet Optimiser (*k*-mer length ranging from 81 to 127), contig scaffolding with Opera^45^ (v1.4.1) and finishing with FinIS^46^ (v0.3).

### Shotgun metagenomic sequencing

DNA from 361 stool samples was extracted using the PowerSoil DNA Isolation Kit (12888, MoBio Laboratories) with modifications to the manufacturer’s protocol. Specifically, to avoid spin-filter clogging, we extended the centrifugation to twice the original duration, and solutions C2, C3 and C4 were doubled in volume. DNA was eluted in 80 µl of Solution C6. Concentration of DNA was determined by Qubit dsDNA BR assay (Q32853, Thermo Fisher Scientific). For library construction, 50 ng of DNA was resuspended in a total volume of 50 µl and was sheared using Adaptive Focused Acoustics (Covaris) with the following parameters: duty factor of 30%, peak incident power of 450, 200 cycles per burst and treatment time of 240 s. Sheared DNA was cleaned up with 1.5× Agencourt AMPure XP beads (A63882, Beckman Coulter). Gene Read DNA Library I Core Kit (180434, Qiagen) was used for end-repair, A-addition and adapter ligation. Custom barcode adapters were used for cost considerations (HPLC purified, double stranded, first strand: 5′ P-GATCGGAAGAGCACACGTCT; second strand: 5′ ACACTCTTTCCCTACACGACGCTCTTCCGATCT) in replacement of Gene Read Adapter I Set for library preparation. Library was cleaned up twice using 1.5× Agencourt AMPure XP beads (A63882, Beckman Coulter). Enrichment was carried out with indexed primers according to an adapted protocol from Multiplexing Sample Preparation Oligonucleotide kit (Illumina). We pooled the enriched libraries in equimolarity and sequenced them on an Illumina HiSeq 2500 sequencing instrument at GIS to generate 2 × 101 base-pair reads, which yielded around 17.7 billion paired-end reads in total and 49 million paired-end reads on average per library.

### Taxonomic and functional profiling

Read quality trimming was performed using famas (https://github.com/andreas-wilm/famas, v0.10,–no-order-check), and microbial reads were identified by mapping and filtering out reads aligned to the human reference genome (hg19) using bwa-mem^47^ (v0.7.9a, default parameters; >90% microbial reads on average). Taxonomic profiling was done using MetaPhlAn^48^ (v2.0, default parameters, filtering taxa with relative abundance <0.1%), and functional profiles were obtained with HUMAnN^49^ (v2.0, default parameters). As a sanity check, we confirmed that species- and genus-level taxonomic profiles were not dominated by taxa that are commonly attributed to reagent or laboratory contamination^50^ (Supplementary File 2). Average-linkage hierarchical clustering of taxonomic profiles was used to group samples with the number of clusters determined using Akaike information criterion. Sample α-diversity was computed using the Shannon diversity index with the vegan library in R. Differential abundance analysis was performed using LEfSe^51^ (v1.0.8), as a non-parametric and conservative approach to identify significantly varying taxa and functions across groups^52^. These results were further validated using Songbird^53^ (v1.0.3; –epochs 10000 –differential-prior 0.5) analysis with Bonferroni-corrected *P* value < 0.05. Abundances of ARGs in the metagenomes was computed using a direct read mapping approach implemented in SRST2 (ref. ^54^) with default parameters and the CARD_v3.0.8_SRST2 database^55^.

### Linear mixed-effects modelling

Linear mixed-effects modelling was conducted using the *lmer* function from the *lme4* package in R. For each model, genus-level Shannon diversity was set as the response variable, with colonization status as the fixed effect and potential confounders (for example, antibiotic usage since last visit, hospitalization status, individual subjects, gender and ethnicity; Supplementary File 1) as random-effect covariates. Residual Shannon diversity values were derived for visualization by subtracting the intercept terms corresponding to random effects.

### SNV analysis

Genome assemblies were aligned to their respective reference genomes using nucmer (v3.23, -maxmatch -nosimplify), and consensus SNVs were called using the show-SNVs function in MUMmer^56^. References for *E. coli* (NC_011750) and *K. pneumoniae* (NC_016845.1) were selected to minimize median distance from isolate genomes. Metagenomic SNVs (consensus and low frequency) were identified on the basis of read mapping using bwa-mem^47^ to the *E. coli* and *K. pneumoniae* references (v0.7.10a; soft-clipped reads and reads with more than three or four mismatches for *K pneumoniae* and *E. coli*, respectively, were filtered out to avoid mismapped reads) and variant calling with LoFreq^57^ (v1.2.1; default parameters). Note that our stringent mapping approach restricts to only reads with >96% identity with the reference and thus will typically exclude mismapping of reads from other genomes. Additionally, genomic regions with frequent ambiguous mappings were identified on the basis of isolate sequencing data and metagenome data from samples without target species as determined from taxonomic classification (>5× coverage with *E. coli* reads on *K. pneumoniae* genome or vice versa). Calls in these regions that match positions where variants were called between isolate reads and reference sequence (allele frequency >95%) were excluded from downstream analysis. The validity of this pipeline was confirmed by noting that very few *K. pneumoniae* SNVs (median 2, mean 5.5) were called genome-wide when analysing metagenomes where taxonomic profiling detected few *K. pneumoniae* reads (ten samples with 107–288 reads). Note that SNVs from such ‘low coverage’ samples are also excluded from further analysis in this study as defined below. To assess the impact of a shared, but potentially divergent, reference on SNV calling, reads were also mapped onto CPE isolate genomes (where available) to call SNVs and compute concordance. Isolate genome-based SNVs were translated to the common reference coordinate system using the UCSC liftover tool^58^ with chain file generated using flo^59^ (-fastMap -tileSize=12 -minIdentity=90).

### Strain analysis

Metagenomic coverage of samples for *E. coli* and *K. pneumoniae* was determined from bwa-mem read mappings using genomeCoverageBed^60^ (v2.25.0). Samples with too low relative abundance for confident identification (<0.1%) were designated as ‘not detected’, while samples with low median read coverage (<8) were designated as ‘low coverage’. Of the remaining samples, those with >90% of the SNVs at or above an allele frequency of 0.9 were designated as ‘one strain’, exhibiting a unimodal distribution as is classically expected in the single haplotype setting^13^ (Supplementary Fig. 5a). A *k*-means clustering approach (based on allele frequency values <0.98, *k* = 2) was used on other samples to identify ‘two strains’ (silhouette score >0.8, indicating good concordance with two clusters for a bimodal distribution) and ‘multiple strains’ cases where there may be more than two clusters (Supplementary Fig. 5a). Note that this analysis was only used to determine strain ‘states’ (Fig. 2), and the corresponding clusters were not used for downstream haplotype analysis. To confirm metagenomic SNV calling quality and strain designations, ‘one strain’ cases were compared with SNVs from corresponding isolates (where available) and noted to have high precision for both *E. coli* and *K. pneumoniae* (>98%; Supplementary Fig. 5b). A first-order Markov model of the transition frequencies between the strain compositions was estimated using the markovchain package in R (ref. ^61^) (maximum likelihood estimator with Laplace smoothing parameter = 1).

### Substrain analysis

SNVs with mean allele frequency >0.9 across timepoints were identified as probably fixed across all strains in a sample. Non-fixed SNVs from ‘one strain’ cases were further annotated for their impact on protein function using SnpEff^62^ (v4.3). The ratio of the rate of non-synonymous (dN) to synonymous (dS) mutations was calculated using the package Biopython.codonalign.codonseq with the ‘NG86’ method.

Leveraging the availability of multiple ‘one strain’ timepoints in some individuals, non-fixed SNV trajectories were clustered to identify co-varying SNVs that may belong to a common substrain background. Specifically, the DBSCAN algorithm in R (ref. ^63^) was used to cluster SNV trajectories in selected individuals with multiple ‘one strain’ timepoints (ε = 0.2, minPts = 2*n* as recommended), and identified clusters were visualized as a sanity check.

### Plasmid analysis

A Mash screen search approach was used with PLSDB^64^ to obtain a list of plasmids that are potentially present in the CPE isolate genomes. The union of all such plasmid sequences was then aligned with isolate genome assemblies to identify plasmids hits with >85% coverage at >95% identity (only alignments >500 bp). Plasmid hits were clustered into groups using hierarchical clustering at 95% identity (hclust function in R, average linkage based on Mash distance^65^), with the longest plasmid serving as a representative. Only plasmids longer than 10 kbp are included in the figure to avoid spurious/redundant matches to shorter plasmids.

### Plasmid conjugation assay

Donor *E. coli* harbouring the pKPC2 plasmid with a kanamycin selection cassette (MG1655) and recipient *K. pneumoniae* strains (ATCC13883) were streaked on selective LBA and incubated overnight at 37 °C. Bacterial colonies were resuspended in LB (1 ml), diluted to OD_600_ 0.5 and mixed in a 1:1 ratio and spotted onto 0.22 µm nitrocellulose membrane (Sartorius) placed on top of LBA (20 µl). After 4 hours of incubation at 37 °C, the bacterial mixture was resuspended in 2 ml of PBS, serially diluted and plated on LBA with appropriate antibiotic selection. Kanamycin (50 μg ml^−1^) and fosfomycin (40 μg ml^−1^) were used for selection of transconjugants. Plates were incubated at 37 °C overnight, and colonies were enumerated. Conjugation frequency was calculated as the total number of transconjugants per total number of recipients.

### Reporting summary

Further information on research design is available in the Nature Research Reporting Summary linked to this article.

### Supplementary information

Supplementary Information Supplementary Figs. 1–8 and Supplementary Table 1.

Reporting Summary

Supplementary Table 1 Sample metadata.

Supplementary Table 2 Taxonomic relative abundance values for various metagenomes based on MetaPhlAn analysis.

Supplementary Table 3 Top ten differentially abundant taxa based on comparisons between metagenomes from distinct community configurations.

Supplementary Table 4 Pathway abundance values for various metagenomes based on HUMAnN analysis.

Supplementary Table 5 Low-frequency, function-altering SNVs in single-strain timepoints.

Supplementary Table 6 Genes containing non-synonymous SNVs that exhibit a large change in allele frequency across pairs of one-strain timepoints in an individual, observed across at least two individuals.

### Source data

Source Data Fig. 1 Ecological-level source data.

Source Data Fig. 2 Strain variations and dynamics source data.

Source Data Fig. 3 Substrain variation for subject 1674-T source data.

Source Data Extended Data Fig. 1 PCoA (with UniFrac distance and Bray–Curtis dissimilarity) source data.

Source Data Extended Data Fig. 2 PCoA correlation study source data.

Source Data Extended Data Fig. 3 Bray–Curtis comparisons to family members source data.

Source Data Extended Data Fig. 4 Pathways analysis source data.

Source Data Extended Data Fig. 5 Enterobacteriaceae relative abundance source data.

Source Data Extended Data Fig. 6 *lacZ* and *lacY* dN/dS values source data.

Source Data Extended Data Fig. 7 *E. coli* substrain variations source data.

Source Data Extended Data Fig. 8 *K. pneumoniae* substrain variations source data.

## Figures and Tables

**Extended Data Table 1:**
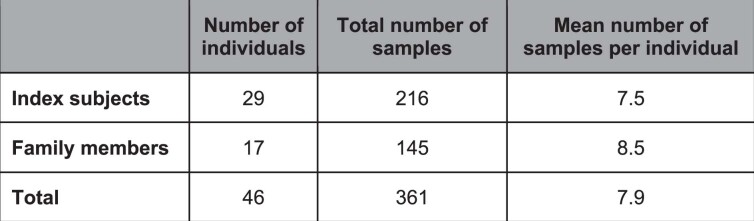
Cohort and sample statistics

Stool samples were collected at recruitment (V00), weekly for 4 weeks (V01-V04), monthly for 5 months (V05-V09) and bimonthly for 6 months (V10-V12) when provided by study participants.

**Extended Data Table 2:**
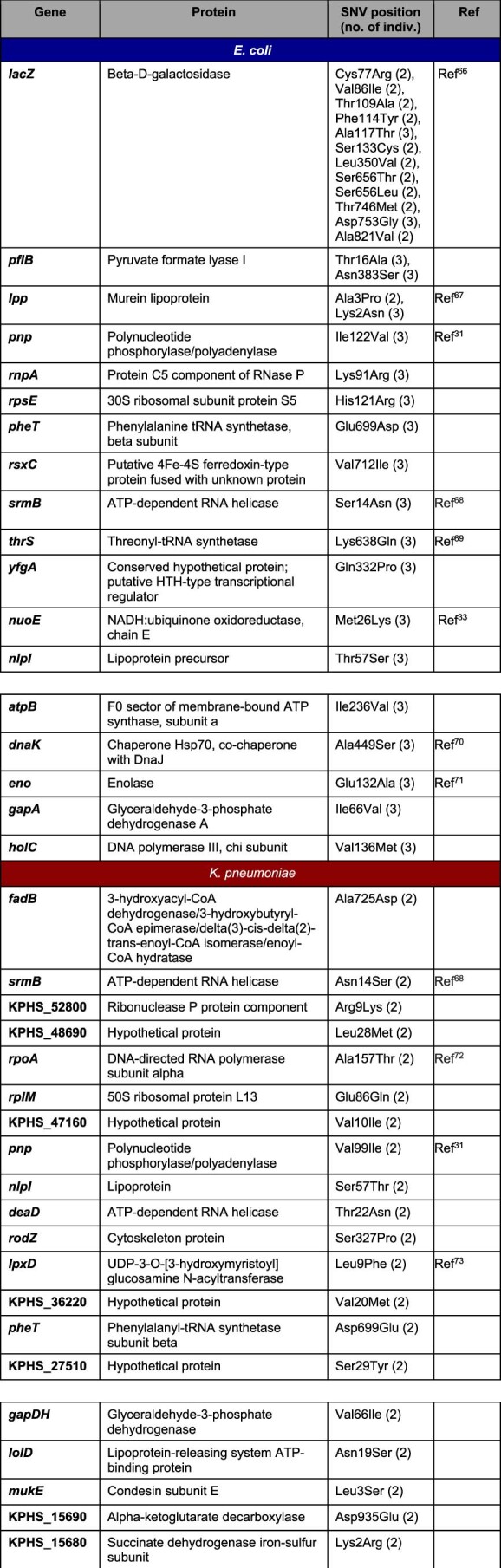
SNVs whose population frequencies varied notably over time recurrently

Genes containing non-synonymous SNVs that exhibit a large (>0.3) change in allele frequency across pairs of one strain time points in an individual, observed across at least two individuals^66–73^.

## Data Availability

Isolate and shotgun metagenomic sequencing data is available from the European Nucleotide Archive (ENA; https://www.ebi.ac.uk/ena/browser/home) under project accession number PRJEB49334. Source data are provided with this paper.

## Code availability

Source code for scripts used to analyse the data are available in a GitHub project at https://github.com/CSB5/CPE-microbiome.
