# Supplementary material for: Long-term ecological and evolutionary dynamics in the gut microbiomes of carbapenemase-producing Enterobacteriaceae colonized subjects
Source: Nat Microbiol. 2022 Sep 15;7(10):1516–24. doi: 10.1038/s41564-022-01221-w (PMC9519440; doi:10.1038/s41564-022-01221-w)
Supplement: Supplementary file 1 — Supplementary Figs. 1–8 and Supplementary Table 1. [file 41564_2022_1221_MOESM1_ESM.pdf]

nature portfolio

https://doi.org/10.1038/s41564-022-01221-w

Supplementary information

Long-term ecological and evolutionary dynamics in the gut microbiomes of carbapenemase-producing Enterobacteriaceae colonized subjects

In the format provided by the authors and unedited

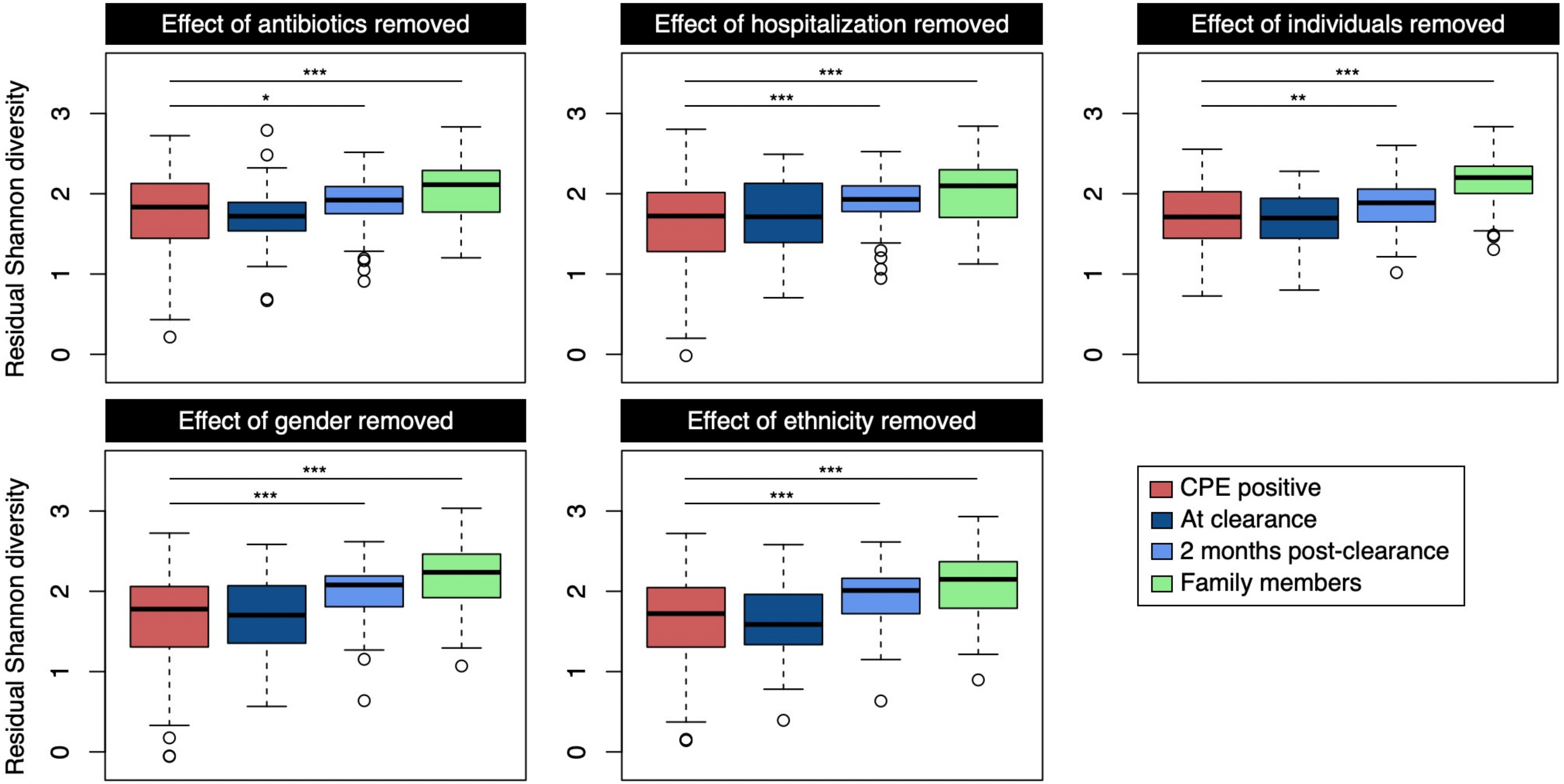

Supplementary Figure 1: Residual Shannon diversity, after subtracting the intercept term due to the random effect in a linear mixed-effects model that treats colonization status as the fixed effect, and the stated covariates as random effects (i.e. antibiotic usage since last visit, hospitalization status, individual subjects, gender and ethnicity; see **Supplementary File 1**). The p-values originate as part of the output from the respective linear mixed effect models, comparing CPE positive timepoints against other groups. (antibiotics, hospitalization: n = 290; individuals, gender, ethnicity: n = 363; respective p-values for CPE positive vs. 2 months post-clearance, and CPE positive vs. family members – antibiotics: 0.100, 1.21×10^{-5}; hospitalization: 0.00215, 3.00×10^{-7}; individuals: 0.0145, 1.94×10^{-4}; gender: 2.85×10^{-5}, $<2\times10^{-16}$; ethnicity: 1.28×10^{-4}, 1.36×10^{-12}) Centre lines in the boxplots represent median values, box limits represent upper and lower quartile values, whiskers represent 1.5 times the interquartile range above the upper quartile and below the lower quartile. *** = p<0.01, ** = p<0.05, * = p<0.1.

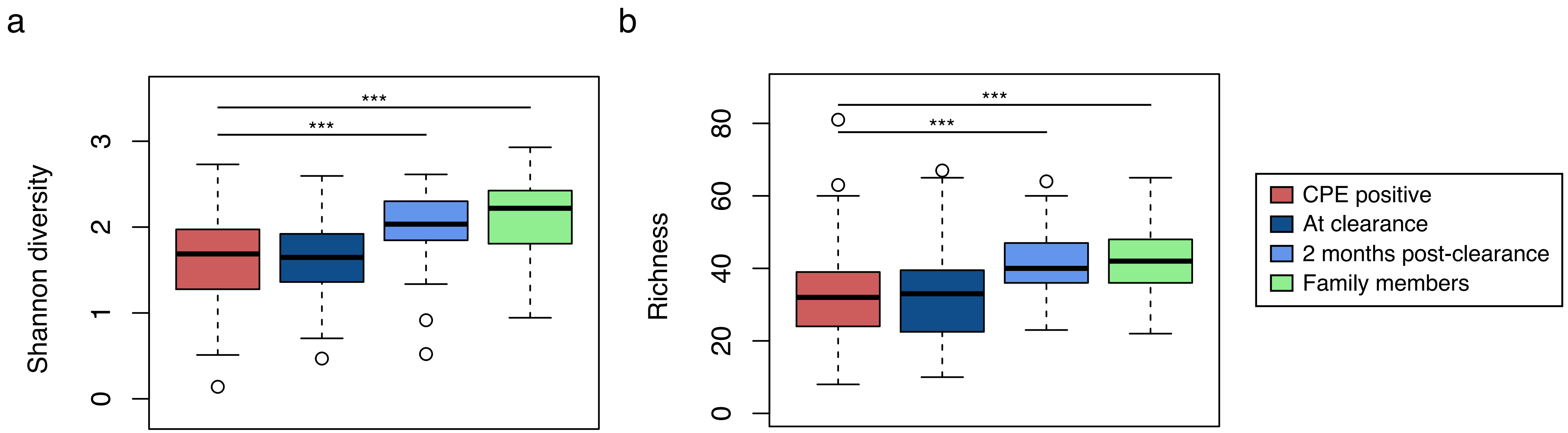

<u>Supplementary Figure 2:</u> (a) Boxplots showing genus-level Shannon diversity distributions for different timepoints for index patients ("CPE positive" = during colonization, "At clearance" = within 1 month of decolonization, and "2 months post-clearance" = time points that were >2 months after decolonization), and all timepoints for family members. This plot is similar to **Figure 1b** but with all *Enterobacteriaceae* counts removed and relative abundances renormalized to 1. (b) Corresponding microbial richness values. (n = 346 timepoints; respective two-sided Wilcoxon rank-sum test p-values for CPE positive vs. 2 months post-clearance, and CPE positive vs. family members – Shannon diversity: 2.20×10^{-6}, 2.20×10^{-14}; richness: 9.97×10^{-7}, 2.64×10^{-13}). Centre lines in the boxplots represent median values, box limits represent upper and lower quartile values, whiskers represent 1.5 times the interquartile range above the upper quartile and below the lower quartile. *** = Wilcoxon rank-sum $p<0.01$ and all other comparisons were not statistically significant.

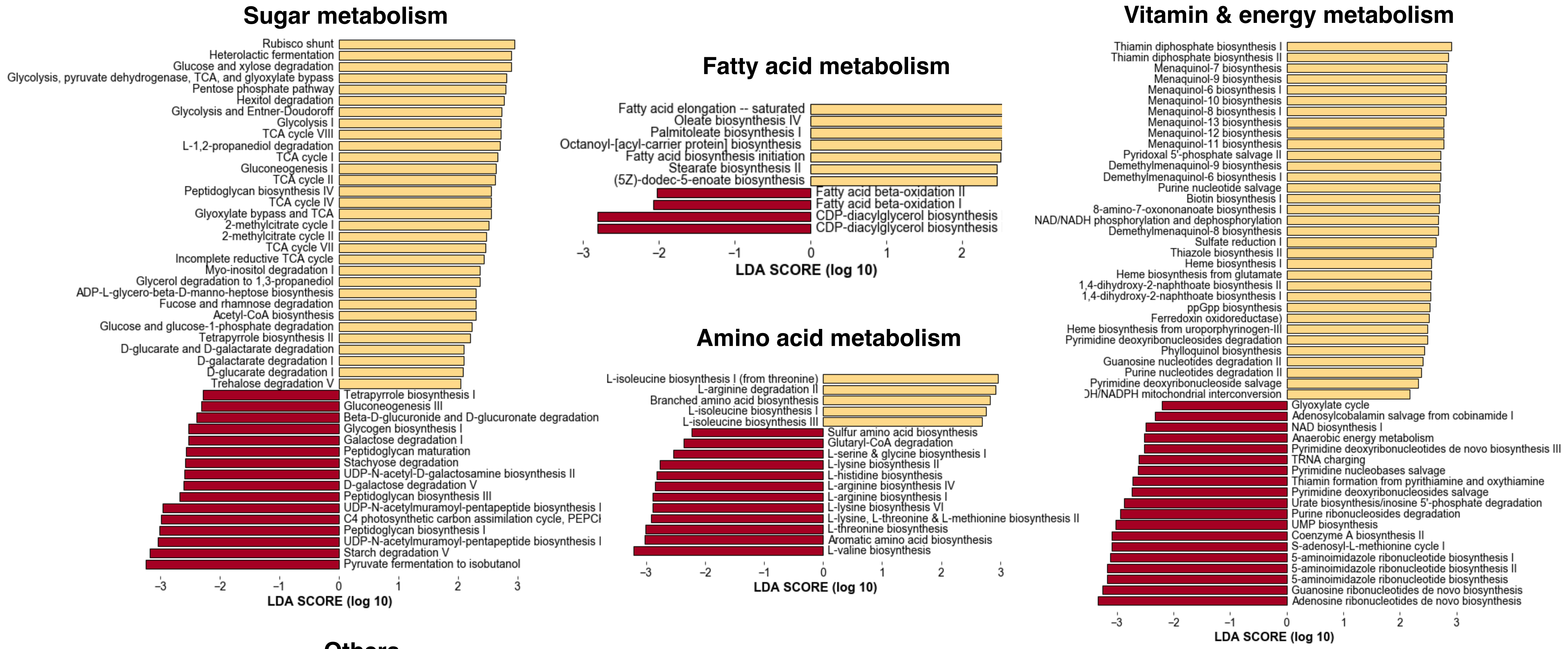

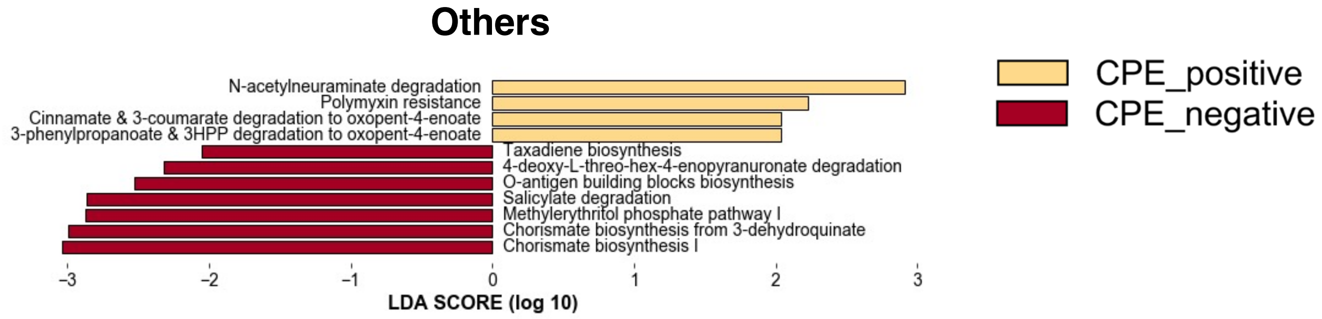

Supplementary Figure 3: Differentially abundant pathways in colonized (CPE positive) and post-decolonization (CPE negative) gut metagenomes (two-sided Wilcoxon rank-sum test, FDR-adjusted p-value<0.05, LDA score>2) after removal of reads assigned to *Enterobacteriaceae* in HUMAnN results and renormalization of abundances.

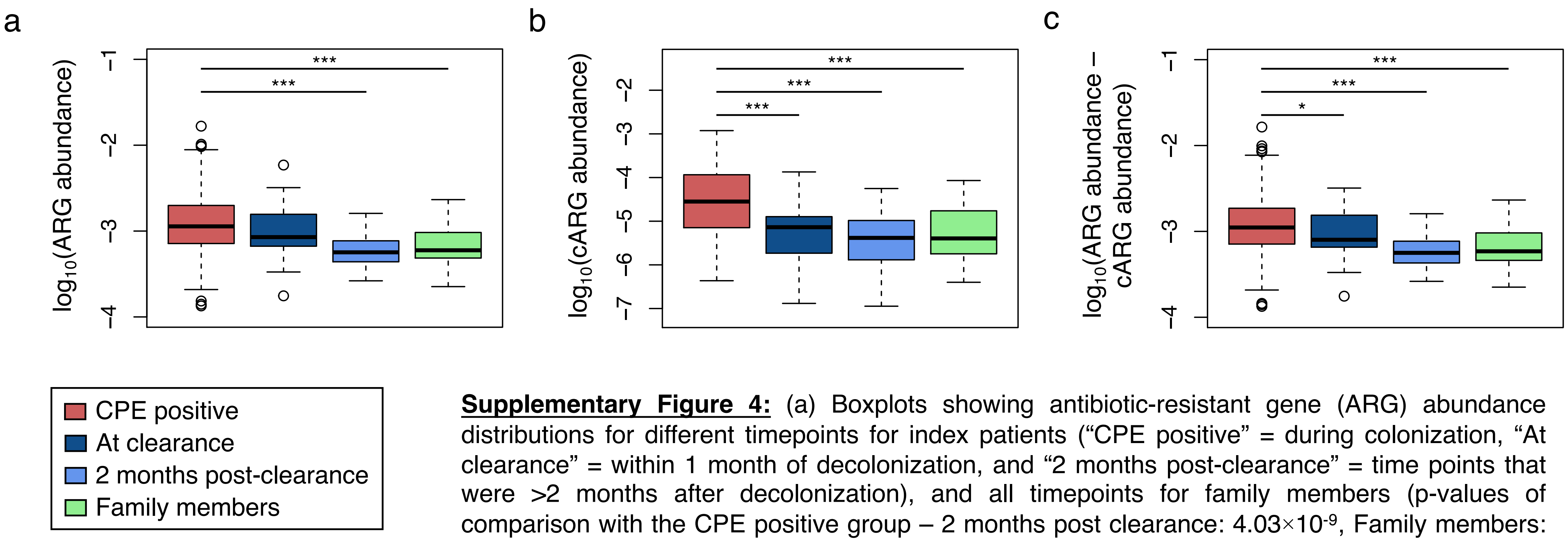

Supplementary Figure 4: (a) Boxplots showing antibiotic-resistant gene (ARG) abundance distributions for different timepoints for index patients ("CPE positive" = during colonization, "At clearance" = within 1 month of decolonization, and "2 months post-clearance" = time points that were >2 months after decolonization), and all timepoints for family members (p-values of comparison with the CPE positive group – 2 months post clearance: 4.03×10^{-9}, Family members: 1.18×10^{-11}). (b) Distribution of corresponding abundance values for carbapenem resistance genes (cARG; ARO:0000020) (p-values of comparison with the CPE positive group – At clearance: 1.49×10^{-5}, 2 months post clearance: 9.02×10^{-9}, Family members: 6.58×10^{-12}). (c) ARG abundance values after subtracting cARG abundances (p-values of comparison with the CPE positive group – At clearance: 0.0731, 2 months post clearance: 8.18×10^{-9}, Family members: 1.96×10^{-11}). *** = Wilcoxon rank-sum $p<0.01$, * = Wilcoxon rank-sum $p<0.1$, and all other comparisons were not statistically significant. n = 345, two-sided Wilcoxon rank-sum test. Centre lines in the boxplots represent median values, box limits represent upper and lower quartile values, whiskers represent 1.5 times the interquartile range above the upper quartile and below the lower quartile.

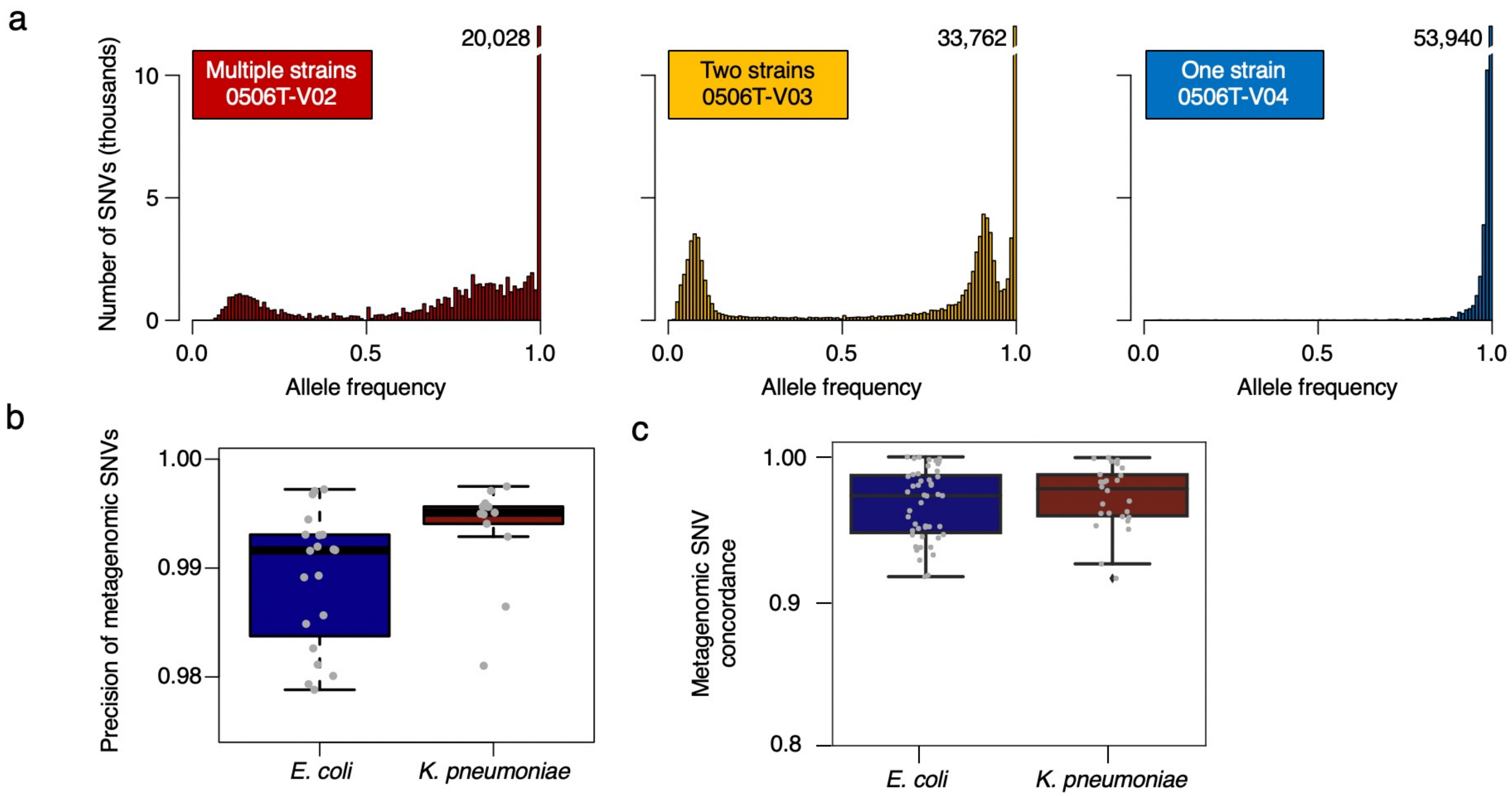

<u>Supplementary Figure 5:</u> (a) Representative allele frequency spectra for samples classified as "one strain", "two strains" and "multiple strains", obtained from 3 consecutive timepoints in subject 0506-T. (b) Boxplots depicting the precision of metagenomic SNVs (One strain, allele frequency ≥0.98) evaluated using SNVs present in corresponding CPE isolates where available (n = 33). (c) Fraction of metagenomic SNVs (all allele frequencies) called using a shared species reference that were recapitulated using sample-specific analysis with CPE isolate references (n = 117). Centre lines in the boxplots represent median values, box limits represent upper and lower quartile values, whiskers represent 1.5 times the interquartile range above the upper quartile and below the lower quartile.

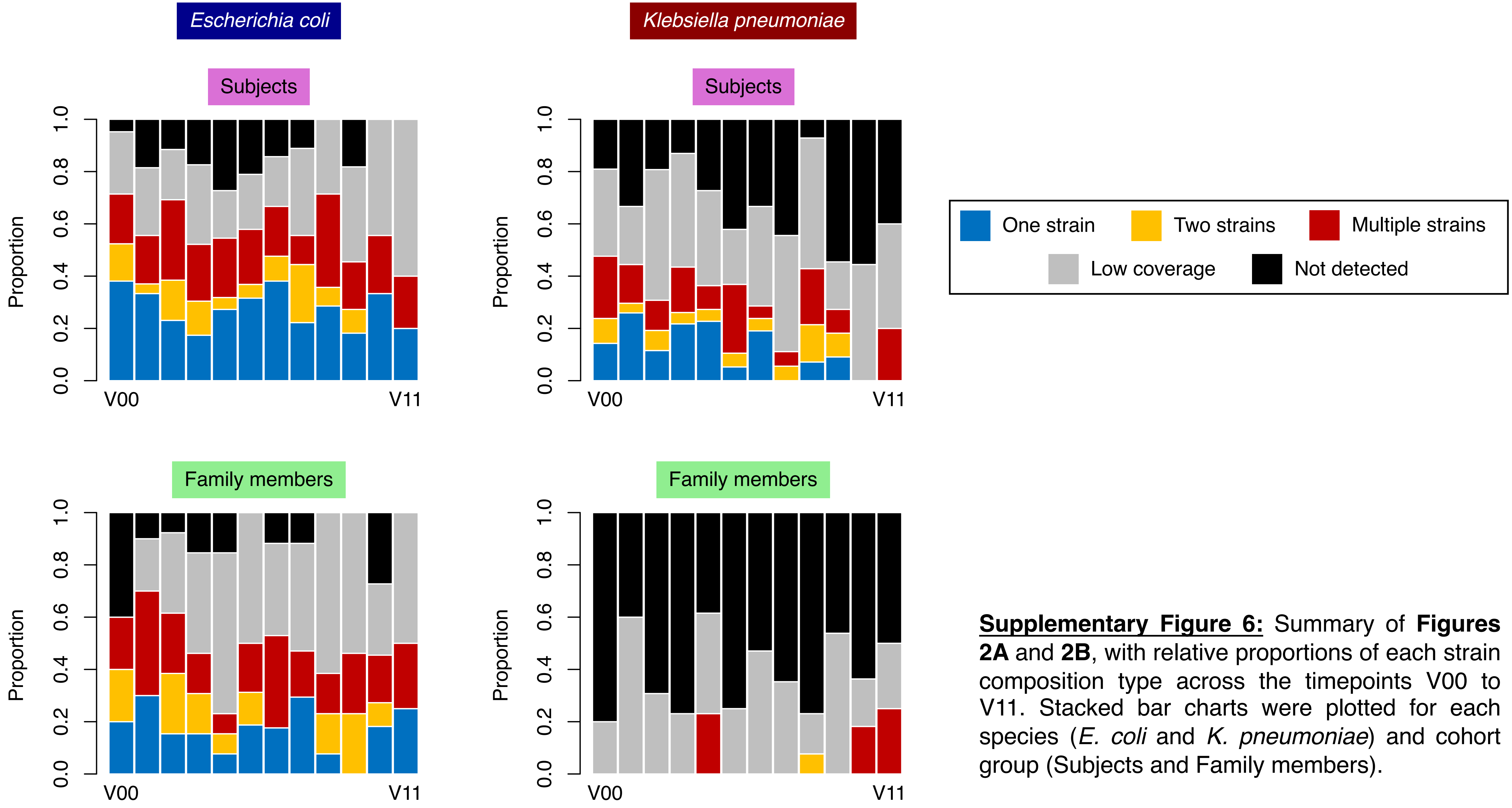

<u>Supplementary Figure 6:</u> Summary of **Figures 2A** and **2B**, with relative proportions of each strain composition type across the timepoints V00 to V11. Stacked bar charts were plotted for each species (*E. coli* and *K. pneumoniae*) and cohort group (Subjects and Family members).

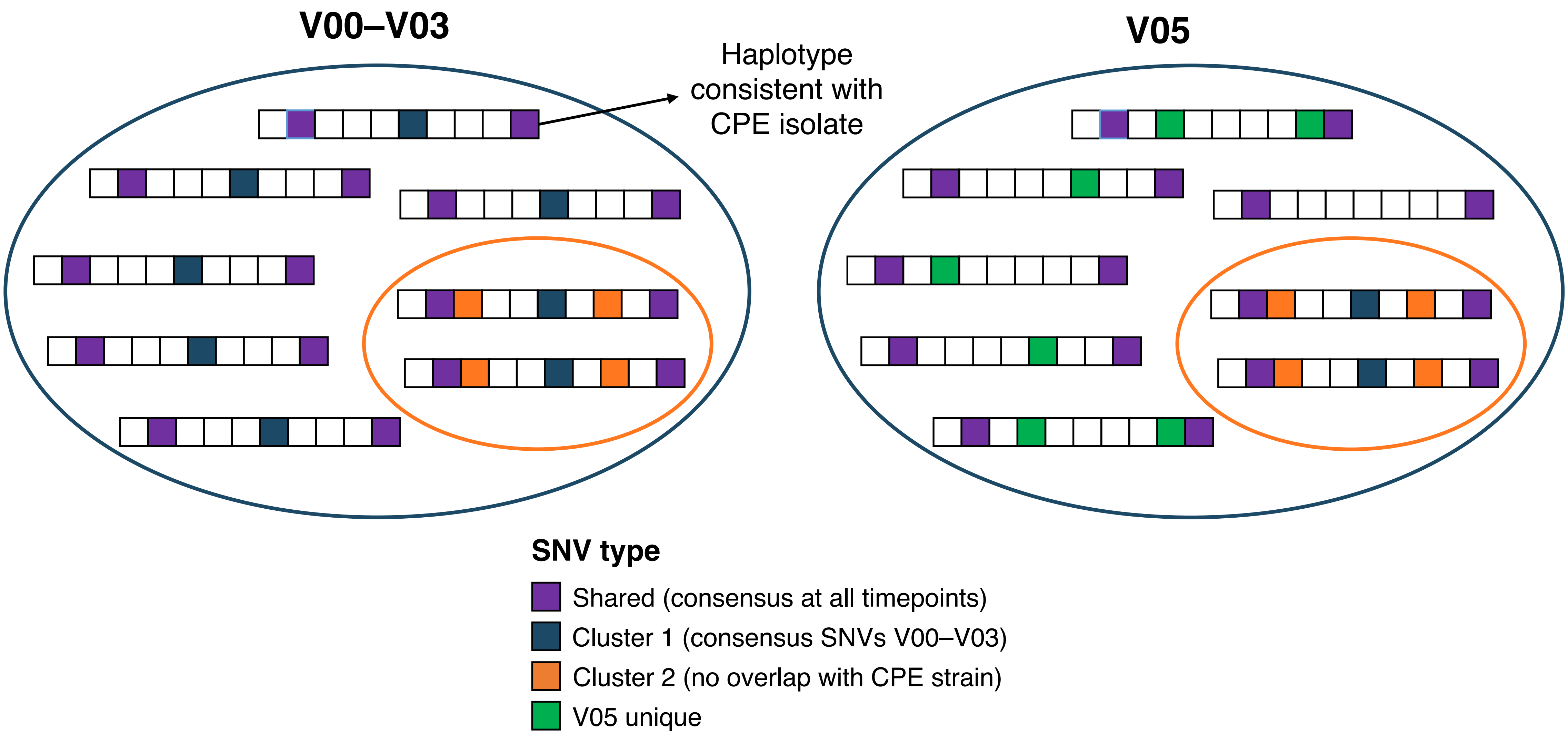

Supplementary Figure 7: Pictorial depiction of haplotype populations consistent with the sub-strain clusters seen in **Figure 3** for *E. coli*. Each strip of boxes represents one haplotype, with white boxes representing reference bases and coloured bases representing SNVs that are part of different time-series clusters from **Figure 3a**. Note that the population on the left depicts the putative state for timepoints V00-V03 and at timepoint V05 there is a loss of the CPE sub-strain to give the population seen on the right.

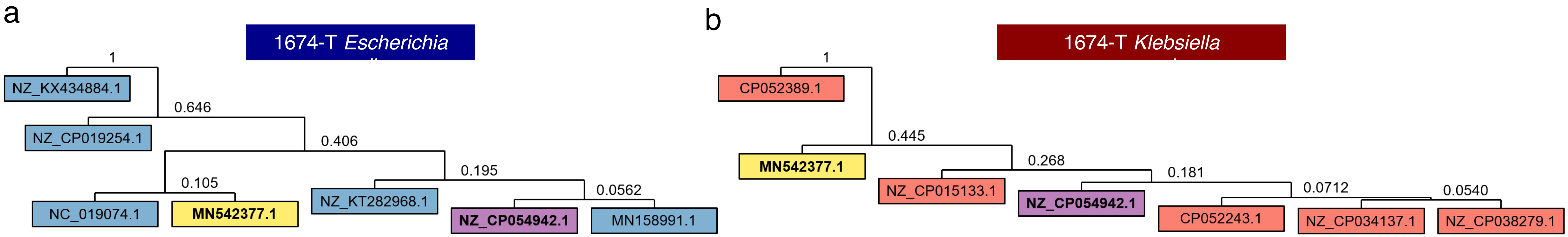

c

Plasmid accession	Plasmid name	Plasmid length (bp)	*E. coli*				*K. pneumoniae*			
			V00	V01	V02	V03	V00	V01	V02	V03
MN542377.1	*K. pneumoniae* strain 494 plasmid pKPC2_sg1	71861	0.979	0.983	0.984	0.979	0.972	0.972	0.972	0.981
NZ_CP054942.1	*E. coli* strain MS6192 plasmid pMS6192B	76661	0.828	0.824	0.809	0.853	0.862	*0.049*	*0.049*	*0.068*
NZ_KX434884.1	*K. pneumoniae* strain KP2442 plasmid pKP2442_4c285	12465	0.866	0.866	0.866	0.866	*0.109*	*0.109*	*0.109*	*0.109*
NZ_CP019254.1	*E. coli* strain 13KWH46 plasmid p13KWH46-4	76784	0.912	0.912	0.911	0.912	-	-	-	-
NC_019074.1	*E. coli* plasmid pHNDD81-1	11744	0.874	0.874	0.874	0.874	*0.640*	*0.553*	*0.553*	*0.611*
NZ_KT282968.1	*E. coli* strain EC012 plasmid pEC012	139622	0.891	0.888	0.884	0.884	*0.074*	*0.069*	*0.067*	*0.102*
MN158991.1	*E. coli* strain TREC8 plasmid pTREC8	118200	0.852	0.860	0.842	0.869	*0.412*	*0.103*	*0.103*	*0.120*
CP052389.1	*K. pneumoniae* strain C17KP0052 plasmid pC17KP0052-1	242988	-	-	-	-	0.997	0.997	0.997	0.997
NZ_CP015133.1	*K. pneumoniae* strain Kpn555 plasmid pKPN-d6b	26450	-	-	-	-	0.907	0.907	0.907	0.993
CP052243.1	*K. pneumoniae* strain E17KP0019 plasmid pE17KP0019-2	246731	*0.106*	*0.107*	*0.108*	*0.107*	0.923	0.898	0.900	0.923
NZ_CP034137.1	*K. quasipneumoniae* strain G747 plasmid pG747_150.8Kb	150833	*0.176*	*0.180*	*0.180*	*0.180*	0.869	0.853	0.858	0.896
NZ_CP038279.1	*R. ornithinolytica* strain WLK218 plasmid pWLK-KPC	35262	*0.303*	*0.310*	*0.311*	*0.303*	0.889	0.855	0.855	0.843

Supplementary Figure 8: Hierarchical clustering of plasmid sequences found in (a) *E. coli* and (b) *K. pneumoniae*, for subject 1674-T across various timepoints (based on Mash distance). Sequences with >95% identity were grouped together and a representative plasmid is shown in the leaves. (c) Table showing the proportion of each representative plasmid that is covered by contigs in various samples. Values in italics and grey indicate proportions that fall below 0.8 and were used to determine the presence, absence pattern shown in **Figure 3h**.

Carbapenemase gene	*E. coli*	*K. pneumoniae*
KPC	17	18
OXA-48	42	31
IMP	6	0
NDM	8	13
IMI	0	1

Supplementary Table 1: Types of carbapenemase genes found in CPE *E. coli* and *K. pneumoniae* isolates.
