# Supplementary material for: Long-term ecological and evolutionary dynamics in the gut microbiomes of carbapenemase-producing Enterobacteriaceae colonized subjects
Source: Nat Microbiol. 2022 Sep 15;7(10):1516–24. doi: 10.1038/s41564-022-01221-w (PMC9519440; doi:10.1038/s41564-022-01221-w)
Supplement: Supplementary file 2 — Reporting Summary [file 41564_2022_1221_MOESM2_ESM.pdf]

nature portfolio

Corresponding author(s): Niranjan Nagarajan
Last updated by author(s): Jul 24, 2022

Reporting Summary

Nature Portfolio wishes to improve the reproducibility of the work that we publish. This form provides structure for consistency and transparency in reporting. For further information on Nature Portfolio policies, see our Editorial Policies and the Editorial Policy Checklist.

Statistics

For all statistical analyses, confirm that the following items are present in the figure legend, table legend, main text, or Methods section.

n/a	Confirmed	
☐	☒	The exact sample size (*n*) for each experimental group/condition, given as a discrete number and unit of measurement
☒	☐	A statement on whether measurements were taken from distinct samples or whether the same sample was measured repeatedly
☐	☒	The statistical test(s) used AND whether they are one- or two-sided<br>*Only common tests should be described solely by name; describe more complex techniques in the Methods section.*
☐	☒	A description of all covariates tested
☐	☒	A description of any assumptions or corrections, such as tests of normality and adjustment for multiple comparisons
☐	☒	A full description of the statistical parameters including central tendency (e.g. means) or other basic estimates (e.g. regression coefficient) AND variation (e.g. standard deviation) or associated estimates of uncertainty (e.g. confidence intervals)
☐	☒	For null hypothesis testing, the test statistic (e.g. *F*, *t*, *r*) with confidence intervals, effect sizes, degrees of freedom and *P* value noted<br>*Give P values as exact values whenever suitable.*
☒	☐	For Bayesian analysis, information on the choice of priors and Markov chain Monte Carlo settings
☒	☐	For hierarchical and complex designs, identification of the appropriate level for tests and full reporting of outcomes
☒	☐	Estimates of effect sizes (e.g. Cohen's *d*, Pearson's *r*), indicating how they were calculated

Our web collection on statistics for biologists contains articles on many of the points above.

Software and code

Policy information about availability of computer code

Data collection	No custom software was used for data collection.
Data analysis	R (v4.0.2), famas (v0.10), bwa-mem (v0.7.9a), MetaPhlAn (v2.0), LEfSe (v1.0.8), nucmer (v3.23), LoFreq (v1.2.1), SnpEff (v4.3), lme4 (v1.1-27.1), genomeCoverageBed (v2.25.0), Velvet (v1.2.10), Opera (v1.4.1), FinIS (v0.3), HUMAnN (v2.0), Songbird (v1.0.3), Custom code available at https://github.com/CSB5/CPE-microbiome

For manuscripts utilizing custom algorithms or software that are central to the research but not yet described in published literature, software must be made available to editors and reviewers. We strongly encourage code deposition in a community repository (e.g. GitHub). See the Nature Portfolio guidelines for submitting code & software for further information.

Data

Policy information about availability of data

All manuscripts must include a data availability statement. This statement should provide the following information, where applicable:

- Accession codes, unique identifiers, or web links for publicly available datasets
- A description of any restrictions on data availability
- For clinical datasets or third party data, please ensure that the statement adheres to our policy

Isolate and shotgun metagenomic sequencing data is available from the European Nucleotide Archive (ENA – https://www.ebi.ac.uk/ena/browser/home) under project accession number PRJEB49334.

Human research participants

Policy information about studies involving human research participants and Sex and Gender in Research.

Reporting on sex and gender	*Use the terms sex (biological attribute) and gender (shaped by social and cultural circumstances) carefully in order to avoid confusing both terms. Indicate if findings apply to only one sex or gender; describe whether sex and gender were considered in study design whether sex and/or gender was determined based on self-reporting or assigned and methods used. Provide in the source data disaggregated sex and gender data where this information has been collected, and consent has been obtained for sharing of individual-level data; provide overall numbers in this Reporting Summary. Please state if this information has not been collected. Report sex- and gender-based analyses where performed, justify reasons for lack of sex- and gender-based analysis.*
Population characteristics	Carriers of carbapenemase-producing Enterobacteriaceae (CPE), and their non-colonized family members as controls. Covariates include gender, age, and ethnicity, but were not the primary focus of our research and findings.
Recruitment	Study participants were recruited from two tertiary care centres in Singapore, based on their status as carriers of carbapenemase-producing Enterobacteriaceae (CPE). We do not identify any self-selection bias in our participant pool.
Ethics oversight	Ethics approval received from the Singapore National Healthcare Group Domain Specific Review Board 74 (NHG DSRB Reference: 2016/00364) prior to commencement.

Note that full information on the approval of the study protocol must also be provided in the manuscript.

Field-specific reporting

Please select the one below that is the best fit for your research. If you are not sure, read the appropriate sections before making your selection.

☒ Life sciences ☐ Behavioural & social sciences ☐ Ecological, evolutionary & environmental sciences

For a reference copy of the document with all sections, see nature.com/documents/nr-reporting-summary-flat.pdf

Life sciences study design

All studies must disclose on these points even when the disclosure is negative.

Sample size	Sample size was determined based on the number of recruited study participants. As stool samples were collected from participants at multiple time points, the eventual number of samples (361) was sufficient for our analyses.
Data exclusions	No data was excluded from the analyses.
Replication	Our data set consists of multiple CPE-colonized participants and their family members, and our findings are generalizable across our participant group. As this is a cohort study, there is no experimentally-based replication.
Randomization	Randomization is not relevant as participants were not assigned to experimental groups.
Blinding	Blinding is not relevant as this study does not involve different treatment groups.

Reporting for specific materials, systems and methods

We require information from authors about some types of materials, experimental systems and methods used in many studies. Here, indicate whether each material, system or method listed is relevant to your study. If you are not sure if a list item applies to your research, read the appropriate section before selecting a response.

Materials & experimental systems

n/a	Involved in the study	
☒	☐	Antibodies
☒	☐	Eukaryotic cell lines
☒	☐	Palaeontology and archaeology
☒	☐	Animals and other organisms
☒	☐	Clinical data
☒	☐	Dual use research of concern

Methods

n/a	Involved in the study	
☒	☐	ChIP-seq
☒	☐	Flow cytometry
☒	☐	MRI-based neuroimaging
